# SAAP-148 Eradicates MRSA Persisters Within Mature Biofilm Models Simulating Prosthetic Joint Infection

**DOI:** 10.3389/fmicb.2021.625952

**Published:** 2021-01-29

**Authors:** Henk Scheper, Julia M. Wubbolts, Joanne A. M. Verhagen, Adriëtte W. de Visser, Robert J. P. van der Wal, Leo G. Visser, Mark G. J. de Boer, Peter H. Nibbering

**Affiliations:** ^1^Department of Infectious Diseases, Leiden University Medical Center, Leiden, Netherlands; ^2^Department of Orthopedic Surgery, Leiden University Medical Center, Leiden, Netherlands

**Keywords:** MRSA, prosthetic joint infection, biofilms, persisters, antimicrobial peptides, SAAP-148, ADEP4

## Abstract

Prosthetic joint infection (PJI) is a severe complication of arthroplasty. Due to biofilm and persister formation current treatment strategies often fail. Therefore, innovative anti-biofilm and anti-persister agents are urgently needed. Antimicrobial peptides with their broad antibacterial activities may be such candidates. An *in vitro* model simulating PJI comprising of rifampicin/ciprofloxacin-exposed, mature methicillin-resistant *Staphylococcus aureus* (MRSA) biofilms on polystyrene plates, titanium/aluminium/niobium disks, and prosthetic joint liners were developed. Bacteria obtained from and residing within these biofilms were exposed to SAAP-148, acyldepsipeptide-4, LL-37, and pexiganan. Microcalorimetry was used to monitor the heat flow by the bacteria in these models. Daily exposure of mature biofilms to rifampicin/ciprofloxacin for 3 days resulted in a 4-log reduction of MRSA. Prolonged antibiotic exposure did not further reduce bacterial counts. Microcalorimetry confirmed the low metabolic activity of these persisters. SAAP-148 and pexiganan, but not LL-37, eliminated the persisters while ADEP4 reduced the number of persisters. SAAP-148 further eradicated persisters within antibiotics-exposed, mature biofilms on the various surfaces. To conclude, antibiotic-exposed, mature MRSA biofilms on various surfaces have been developed as *in vitro* models for PJI. SAAP-148 is highly effective against persisters obtained from the biofilms as well as within these models. Antibiotics-exposed, mature biofilms on relevant surfaces can be instrumental in the search for novel treatment strategies to combat biofilm-associated infections.

## Introduction

Yearly over one million prosthetic joints are implanted in patients in the United States. Prosthetic joint infection (PJI) is a severe complication occurring in 1–3% of patients and has a high economic burden on health care systems. Most PJIs are caused by staphylococci (Tande and Patel, 2014; Zeller et al., 2018). Treatment of patients with an acute PJI consists of thorough surgical debridement of the implant and the infected tissue around it, followed by 6–12 weeks of antibiotic therapy. Nevertheless, failure rates for this treatment strategy are considerable, ranging from 10 to 45% in some of the largest studies (Lora-Tamayo et al., 2013, 2017). An important cause of treatment failure is the formation of a biofilm on the surface of the implant. A biofilm is formed by bacteria that, after adherence to the implant, form a matrix of extrapolymeric substances (EPS) that protect bacteria against the actions of antibiotics and effectors of host’s immune systems (Lewis, 2010). Within biofilms bacteria may switch phenotypically to a metabolically inactive, non-dividing, dormant state, called persisters (Balaban et al., 2004; Lewis, 2010). Persisters are defined as metabolically inactive, dormant bacteria that survive lethal concentrations of antibiotics without induction of resistance. The formation of persisters is triggered by stress factors like lack of nutrients and exposure to antibiotics (Harms et al., 2016). Persisters within biofilms are tolerant to antibiotic therapy, which contributes to PJI treatment failures (Conlon et al., 2013). Based on these considerations, innovative anti-biofilm and anti-persister treatment strategies are urgently needed. For evaluation of such candidates an *in vitro* biofilm model that approximates a PJI as closely as possible is instrumental.

Antimicrobial peptides are considered promising candidates to combat biofilm-associated infections. For instance, the human cathelicidin LL-37 has broad-spectrum antibacterial activities, including antibiofilm activity, together with immune modulating capabilities (van der Does et al., 2010). SAAP-148, a synthetic peptide based on LL-37, has shown to be more effective in eradicating bacteria than LL-37 (de Breij et al., 2018). Acyldepsipeptide 4 (ADEP4) activates bacterial proteases in an ATP-independent manner resulting in cell death. In combination with rifampicin, ADEP4 eradicates biofilms in a mouse model with a chronic *S. aureus* infection (Conlon et al., 2013). Pexiganan, an analog of magainin isolated from the skin of the African clawed frog, exhibited *in vitro* broad-spectrum antibacterial activity (Ge et al., 1999; Haisma et al., 2016).

For the current study we developed an *in vitro* biofilm model approximating a PJI as closely as possible. With this model the efficacy of four promising antimicrobial peptides on persisters and bacteria in other growth modes in mature biofilms was assessed.

## Materials and Methods

### Antibiotics and Antimicrobial Peptides

Ciprofloxacin hydrochloride (Sigma-Aldrich, PHR 1044-1G) and rifampicin (Sigma-Aldrich, R3501-250 mg) at concentrations corresponding to 10x the minimal bactericidal concentration (MBC) for MRSA LUH14616 were used (1.28 mg/mL ciprofloxacin, 10 μg/mL rifampicin). The MBC was defined as the lowest concentration that killed 99.9% of the bacteria compared to untreated control bacteria. SAAP-148 (LKRVWKRVFKLLKRYWRQLKKPVR), LL 37 (LLGDFFRKSKEKIGKEFKRIVQRIKDFLRNLVPRTES), and pexiganan (GIGKFLKKAKKFGKAFVKILKK), all N-terminal acetylated, C-terminal amidated, were synthesized by solid phase strategies on an automated multiple peptide synthesizer (SyroII, MultiSyntech, Witten, Germany) as described elsewhere (Nell et al., 2006; Haisma et al., 2014; de Breij et al., 2018). The molecular mass of the peptides was confirmed by mass spectrometry and the purity of the peptide exceeded 95%, as determined by reverse phase high-performance liquid chromatography. The lyophilized peptides were stored at −20°C until use. The Clp protease activator acyldepsipeptide 4 (ADEP4) was purchased from ABGENT, a WuXi AppTec company (China); the purity of this commercial peptide was >98%. Peptide stocks were stored in 0.01% acetic acid (pexiganan), dimethyl sulphoxide (ADEP4), and milliQ (SAAP-148 and LL-37). For experiments, peptide stocks were diluted in phosphate-buffered saline (PBS) to the desired concentrations.

### Methicillin-Resistant *Staphylococcus aureus*

Methicillin-resistant *Staphylococcus aureus* (MRSA) LUH14616 sequence type 247, was collected by a nasal swab from a patient without an infection. It was preserved in nutrient broth supplemented with 20% glycerol at −80°C. Prior to experiments, inocula from the frozen stocks were grown overnight at 37°C on sheep blood agar plates (BioMerieux). Thereafter, bacteria were cultured to mid-log phase in tryptic soy broth for 2.5 h at 37°C. Finally, the bacteria were harvested by centrifugation (1,000 × *g* for 10 min) and then resuspended in PBS to the desired inoculum concentration (10^7^ CFU/mL), based on the optical density at 600 nm.

### *In vitro* Biofilm Model Simulating Biofilm Associated Infection

**FIGURE 1:**
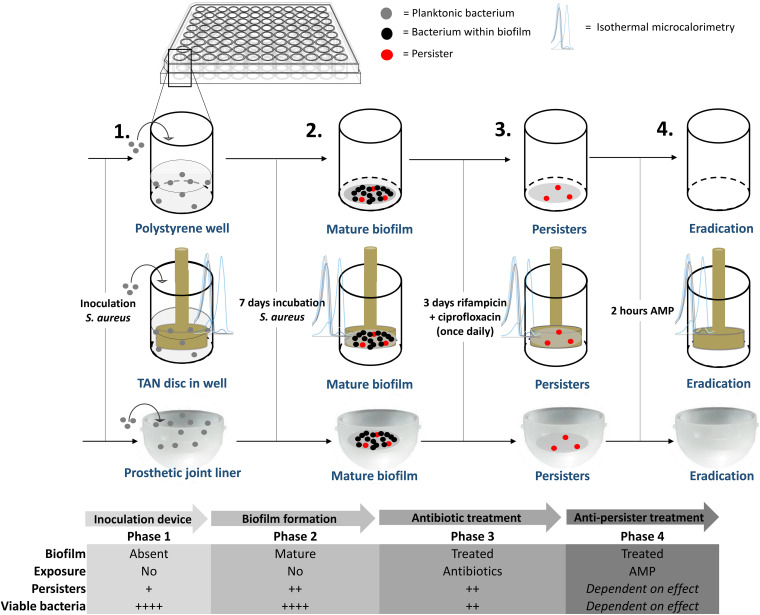
Overview of the mature, antibiotics-exposed biofilm model. Briefly, methicillin-resistant *Staphylococcus aureus* (MRSA) were inoculated into a well, on a metal implant device within a well and into a sterile acetabular hip liner, incubated for 7 days, exposed to rifampicin and ciprofloxacin and subsequently exposed to antimicrobial peptide. AMP: antimicrobial peptide. Estimated proportion of persisters/viable bacteria: +, < 0.1%, ++, > 0.1% and < 1%; ++++, 10–100%.

Mid-log phase MRSA were diluted to 10^7^ CFU/mL in brain heart infusion (BHI) medium. Next, 100 μl of this suspension were cultured in 96-wells polystyrene plates covered with breathable seals and incubated at 37°C for 7 days in a humidified environment. Thereafter, the medium was removed and the wells were washed twice with PBS (140 mM NaCl and pH 7.4). Next, 100 μL of fresh BHI medium containing ciprofloxacin and rifampicin, both 10x MBC, was carefully added to each well in order not to disrupt the biofilm. The medium containing antibiotics was refreshed daily for 72 h. In the second model TAN disks (consisting of titanium7%-aluminium6%-niobium; ISO5832/11) were inserted in the 96-well plates and mature biofilms were developed on these metal disks using the protocol as above. A third model comprised of bacterial biofilms developed on the bottom of the cup of a prosthetic hip liner. Twelve ultra-high-molecular-weight polyethylene acetabular cups were provided by Waldemar Link GmbH & Co., KG (Germany). During formation of the biofilm and antibiotic exposure, the liner was covered with aluminium foil to prevent contamination and dehydration of the biofilm. Liners were re-used due to the limited number of liners provided. Prior to re-use, the liner was, after rinsing with 70% ethanol, submerged in 70% ethanol overnight after which the liner was rinsed again with 70% ethanol and autoclaved thereafter. The experiments with the liners were done twice. In the first dose-finding experiment liners were tested per increasing SAAP-148 concentration. Thereafter, the experiment was repeated for the concentrations with the highest effectivity in the first experiment. A schematic overview of the different models is provided in Figure 1.

### Assessment of the Effects of Antimicrobial Peptides on Persisters in Antibiotics-Exposed Mature Biofilms

The activity of antimicrobial peptides against bacteria, i.e., persisters and possibly other bacterial subpopulations with a long lag time, in antibiotics-exposed mature biofilms was assessed by exposure of the biofilms to increasing concentrations of antimicrobial peptides in PBS for 2 or 24 h after removal of the supernatant. Prior to and at the indicate interval after exposure to the peptide, biofilms were sonicated in PBS and the number of surviving bacteria was determined microbiologically. In case of complete eradication bacterial plates were inspected again after 5 days in the incubator for possible regrowth of persisters and/or bacteria with a long lag time. To assess the direct effects of the peptides on these bacterial subpopulations, antibiotics-exposed mature biofilms from multiple wells were sonicated, pooled and diluted in PBS and then exposed to increasing concentrations of antimicrobial peptides. The possibility that not all bacteria could be obtained from biofilms by sonication was investigated microbiologically and we did not find viable bacteria (even up to 5 days of maintaining the bacterial plates in the incubator) remaining in the wells. In addition, the viability of bacteria was also not affected by the sonication procedure used in these experiments.

### Isothermal Microcalorimetric Assay

Isothermal microcalorimetry (IMC) was used to monitor heat flow (μW) by MRSA in four different stages during the formation of antibiotics-exposed mature biofilms on TAN disks in real time for 30 h using a Calscreener (Symcel Sverige, Spånga, Sweden). BHI broth was used as a reference to calibrate the Calscreener. Mature biofilms and antibiotics-exposed mature biofilms were developed in this model as described above. After removal of the supernatant by two washes with PBS the inserts containing the biofilms were transferred to metal microcontainers and then exposed to 100 μL of BHI with or without 51.2 μM SAAP-148 peptide. In addition, 100 μL of 1 × 10^7^ CFU planktonic MRSA/mL was transferred to metal microcontainers. Furthermore, microcontainers with 100 μL of BHI served as a control. All metal microcontainers were maintained in the Calscreener for 4 days at 37°C for continuous monitoring of the heat production by the various bacterial populations. At the end of the experiments the microcontainers were sonicated for 10 min and the number of persisters was determined microbiologically and cultured for 5 days afterward.

### Statistical Analysis

The non-parametric Mann–Whitney *U* test was performed to determine statistical significance when comparing medians of antibiotics and/or antimicrobial peptide treated biofilms using GraphPad prism (GraphPad software, La Jolla, CA, United States). *P* values ≤ 0.05 were considered to be statistically significant different.

## Results

### Effect of Antibiotics on Bacteria Within Mature Biofilms on Polystyrene

**FIGURE 2:**
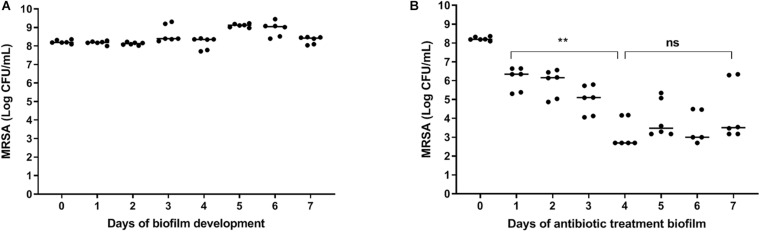
| Effect of antibiotics on bacteria within mature biofilms on polystyrene. **(A)** The number of bacteria within biofilms on 96-well polystyrene plates remained constant for 7 days. **(B)** Exposure of a seven-day mature biofilm on 96-well polystyrene plates to rifampicin/ciprofloxacin daily for 3 days, significantly reduced the bacterial load (*p* = 0.002). Prolonged exposure to rifampicin/ciprofloxacin did not result in further reduction of bacterial counts. The solid line denotes the median log CFU/mL. *n* = 3 experiments, each in duplicate. ns, not significant.

A constant bacterial load of 8 log CFU/ml was present during the seven days of biofilm maturition (Figure 2A). Results revealed a time-dependent reduction in bacterial counts in seven-day mature antibiotics-exposed MRSA biofilms on polystyrene plates with a maximum 4 log reduction at day three-four (Figure 2B), indicating that the surviving bacteria were antibiotic-tolerant. Therefore, antibiotic exposure of mature biofilms for more than 3 days was considered sufficient for persister enrichment.

### MRSA in Antibiotics-Exposed Mature Biofilms Are Dormant

**FIGURE 3:**
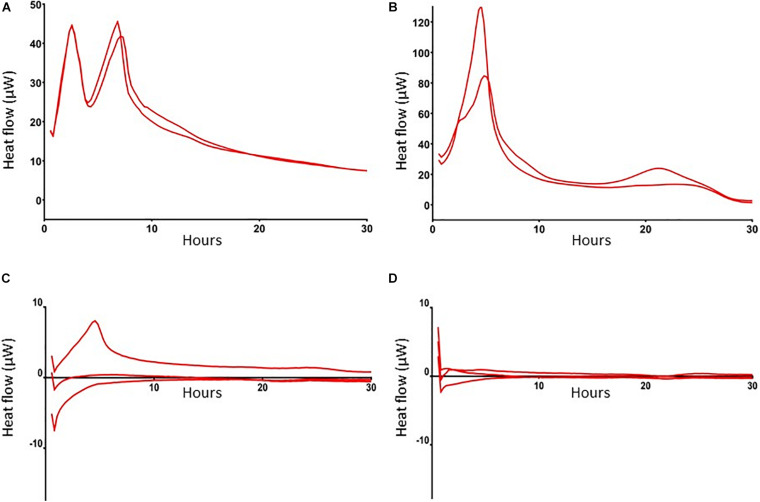
Heat flow by MRSA in log phase, in mature biofilms and in mature, antibiotics-exposed biofilms with and without exposure to SAAP-148. Heat flow by 1 × 10^6^ log phase MRSA **(A)**, >5 × 10^8^ MRSA in a mature biofilm **(B)**, 1 × 10^4^ persisters in antibiotics-exposed biofilms upon exposure to BHI broth **(C)**, and antibiotics-exposed mature biofilms to BHI supplemented with 51.2 μM SAAP-148 **(D)**. Log-phase MRSA, MRSA in mature biofilms and antibiotics-exposed biofilms were maintained in microcontainers in a Calscreener during 30 h. There was no heat flow in the persister subpopulation apart from a small peak after addition of BHI **(C)**. Persisters exposed to SAAP-148 did not display any detectable heat flow **(D)**. Results are from two replicates of a representative experiment (*n* = 2–3 experiments).

To further characterize these antibiotic-tolerant bacteria their metabolic activity was measured by isothermal calorimetry. Results revealed that heat flow of these bacteria in antibiotics-exposed, mature biofilms was almost zero (Figures 3A–C), indicating that these bacteria are metabolically inactive, i.e., persisters. Of note, an initial modest peak of 7 μW upon incubation of these cells with BHI confirms their ability to revive. For comparison, we also assessed heat flow by planktonic MRSA and bacteria in mature biofilms. Results revealed two peaks in the heat flow curve of planktonic bacteria: the first peak occurred after 2.5 h of incubation and the second peak at 6–8 h (Figure 3A). Thereafter, heat flow dropped to a value of approximately 10 μW, probably due to the evolution to stationary phase bacteria with less metabolic activity. MRSA within mature biofilms supplemented with BHI (bacterial load > 5 × 10^8^ MRSA) showed a peak in heat production around 6 h after which the level decreased to a continuous level of 10 μW, which was equal to heat production by stationary phase bacteria (Figure 3B), indicating that heat production by bacteria within a biofilm is considerably less than by mid log phase bacteria.

### Effect of SAAP-148, ADEP4, LL-37, and Pexiganan on MRSA Persisters

**FIGURE 4:**
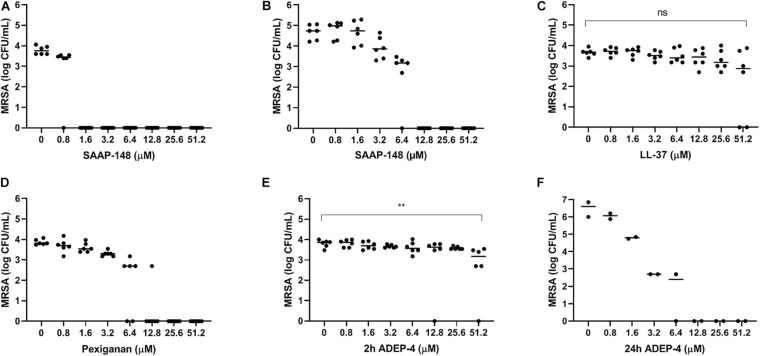
Effect of SAAP-148, ADEP4, LL-37, and pexiganan on MRSA originating from antibiotics-exposed seven-day mature biofilms. SAAP-148 resulted in complete eradication of bacteria sonicated from antibiotics-exposed seven-day mature biofilms on polystyrene at doses ≥1.6 μM **(A)**. Exposure of the intact biofilm without sonication to SAAP-148 resulted in complete eradication at doses ≥12.8 μM **(B)**. Pexiganan resulted in complete eradication of sonicated bacteria at doses ≥6.4 μM **(C)**. Acyldepsipeptide 4 (ADEP4) **(D)** reduced the bacterial counts by 1 log. Exposing the bacteria for 24 h to ADEP4 **(E)** resulted in compete eradication at doses ≥12.8 μM. Human cathelicidin LL-37 **(F)** reduced the bacterial counts by 1 log. Solid lines denote the median log CFU/mL. Figures 3A–E: *n* = 3 experiments (each in duplicate) Figure 3F: *n* = 1 experiment in duplicate. ns, not significant.

To select the most promising antimicrobial peptide, the direct effect of SAAP-148, ADEP4, LL-37, and pexiganan on persisters obtained from antibiotics-exposed, mature MRSA biofilms on polystyrene plates was assessed (Figure 4). Within 2 h SAAP-148 (at doses ≥ 1.6 μM; Figure 4A) and pexiganan (at doses ≥ 12.8 μM; Figure 4B) eradicated all bacteria, whereas bacterial counts were reduced by LL-37 (Figure 4C) and ADEP4 (Figure 4D). In agreement with the expectation that ADEP4 requires more time to exert its effects, we found that at 24 h of exposure all persisters were eliminated by ADEP4 (at doses ≥ 12.8 μM; Figure 4E). Of note, bacterial samples obtained after exposure to the peptides were cultured up to 5 days to ascertain that all persisters were killed. In addition, SAAP-148 was also highly effective in eliminating persisters residing in antibiotics-exposed, mature biofilms with complete eradication seen at ≥1.6 μM (Figure 3F). Based on these data SAAP-148 was selected for further experiments.

### Effect of SAAP-148 on MRSA Persisters Obtained From and Residing in Antibiotics-Exposed Mature Biofilms on TAN Disks

**FIGURE 5:**
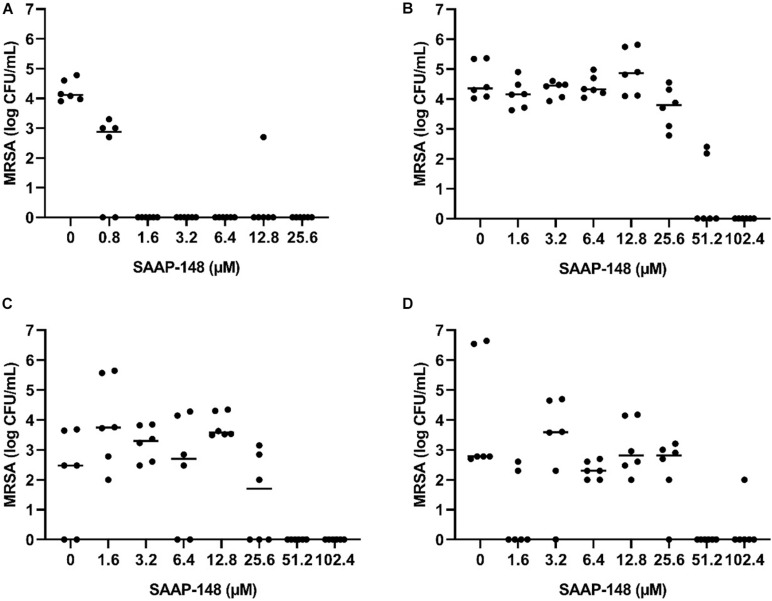
Effect of SAAP-148 on MRSA persisters in antibiotics-exposed mature biofilms on TAN disks. Bacteria obtained by sonication from antibiotics-exposed mature biofilms on TAN disks were exposed for 2 h to SAAP-148. This resulted in eradiation of the bacteria at SAAP-148 doses of ≥1.6 μM **(A)**. SAAP-148 dose-dependently reduced bacterial counts in intact biofilms (without sonication) on TAN disks at doses ≥51.2 μM **(B)**. Prolonging the exposure of the biofilms to SAAP 148 to 24 h resulted in similar eradication of bacteria at doses ≥51.2 μM **(C)**. Addition of SAAP 148 for 24 h during an additional fourth day of antibiotic exposure on the intact biofilm also resulted in eradication of bacteria **(D)**. Solid lines denote the median log CFU/mL (*n* = 3 experiments, each in duplicate).

Next, seven-days mature MRSA biofilms were produced on TAN disks and then exposed for 3 days to rifampicin and ciprofloxacin. SAAP-148 eliminated all persisters obtained from these antibiotics-exposed mature MRSA biofilms in a dose-dependent fashion with complete eradication already seen at ≥1.6 μM (Figure 5A). Exposure of the persisters residing in antibiotics-exposed mature biofilms to SAAP-148 also resulted in complete eradication, but at higher doses (≥51.2 μM; Figure 5B). Prolonged exposure of the persisters in biofilms to SAAP-148 did not improve the efficacy of the peptide (eradication at doses ≥ 51.2 μM; Figure 5C). To rule out the possibility that SAAP-148 was in fact effective against persisters which became metabolically active again after quitting antibiotic therapy, the experiment was repeated with addition of the antibiotics together with SAAP-148 on the fourth day of antibiotic exposure. This also resulted (in five out of six experiments) in elimination of all biofilm-embedded bacteria from a dose of 51.2 μM (Figure 5D). In agreement, calorimetry showed that SAAP-148 reduced heat production of bacteria residing in the biofilm on TAN disks to undetectable levels (Figure 3D). Together, these data indicate that higher doses of SAAP-148 are required to eliminate bacteria within the mature biofilm than when directly in contact with the persisters.

### Effect of SAAP-148 on MRSA Biofilms Formed on a Polyethylene Insert of a Prosthetic Hip Joint

**FIGURE 6:**
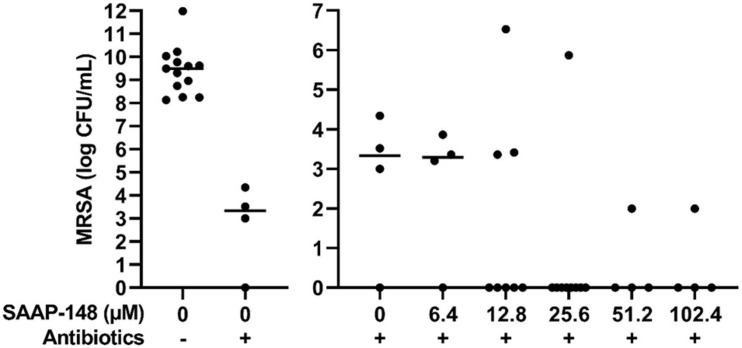
Effect of SAAP-148 on mature antibiotics-exposed MRSA biofilms on an acetabular hip liner. From a SAAP-148 dose of 25.6 μM, bacteria were eradicated from the majority of liners. This experiment was performed twice. In the second experiment, more liners were used for the concentrations with the highest effectivity in the first experiment. Controls and 6.4 μM: *n* = 4 liners; 12.8 μM: *n* = 8 liners; 25.6 μM: *n* = 9 liners; 51.2 and 102.4 μM: *n* = 4 liners. Outliers were present in both experiments. Antibiotics-, before exposure to antibiotics; Antibiotics+, after exposure to antibiotics. Solid lines denote the median log CFU/mL.

To simulate a PJI more closely, the effect of different SAAP-148 concentrations was assessed on persisters in antibiotics-exposed, mature biofilms on sterile acetabulum liners of a hip prosthesis. Results revealed eradication of the bacteria by peptide at all concentrations ≥25.6 μM except for three outliers (Figure 6). The results indicate that SAAP-148 is also effective against persisters in mature biofilms on acetabular hip liners.

## Discussion

### Importance of an *in vitro* Mature Biofilm Model Simulating PJI and Other Foreign Material Infections

As antibiotic treatment of PJI often fails novel agents that eradicate persisters in mature biofilms are urgently needed. In this study we compared the anti-persister activities of synthetic antimicrobial peptides as potential candidates. For this purpose, we first developed innovative *in vitro* models simulating a PJI. These models were based on the following considerations. First, in most clinical biofilm-associated infections like PJI a mature biofilm has developed. The heterogeneous biofilm structure with extracellular polymers substances, eDNA and proteins makes drug penetration more difficult (Wolcott et al., 2010). Also, the subpopulation of persisters in mature biofilms is denser than in immature biofilms (that are often used in biofilm studies) due to antibiotic treatment and nutrient starvation. To avoid outcomes that may not be optimal for translation to clinical biofilm-associated infections, we used seven-day matured MRSA biofilms followed by 3 days of antibiotic exposure. Second, we developed the biofilms on acetabular hip liners and metal alloys (titanium, niobium, and aluminum) that are used in prosthetic joints. Third, to avoid awakening of persisters, exposure of the persisters to antimicrobial peptides is initiated immediately after termination of antibiotic exposure. Finally, we ruled out late regrowth of surviving persisters in biofilms after SAAP-148 exposure by inspecting the bacterial agar plates after 5 days in the incubator. These *in vitro* models can be used to screen other anti-biofilm and anti-persister agents, although limitations in simulating PJI should be taken into account, such as the absence of host cells and inflammatory mediators.

### Antibiotic Tolerance of Persisters to Rifampicin and Ciprofloxacin

We exposed mature biofilms to high doses of rifampicin and ciprofloxacin. These antibiotics are widely used as treatment for staphylococcal PJI, penetrate well in biofilms, and reduce bacterial counts within biofilms significantly (Zimmerli and Sendi, 2019). We found that the antibiotics reduced the bacterial load in mature biofilms by >99.9% with the remaining bacteria displaying tolerance for high doses of rifampicin and ciprofloxacin. Microcalorimetry confirmed the dormant state of these antibiotics-tolerant bacterial cells as well as their ability to revive upon addition of bacterial growth medium. A limitation of microcalorimetry is its lower limit of detection being approximately 1 × 10^4^ bacteria (Braissant et al., 2010), which is close to the number of persisters in the mature biofilms. Also, the measured heat flow is the sum of all chemical and physical processes that take place within the bacterial community. Obviously, additional and more sensitive methods, for example transcriptome analysis, cryo-electron microscopy and/or measurement of ATP levels in bacteria within biofilms, should be implicated to further characterize the persisters. Nevertheless, we can conclude that substantial numbers of persisters are present in the current antibiotics-exposed, mature biofilms. We cannot exclude that bacteria with a long lag time or small colony variants (SCVs) also survived antibiotic exposure. SCVs differ from the normal phenotype in their small colony size and reduced growth rate. However, complete elimination of all bacterial cells that were not affected by the antibiotics indicates that these SCVs, if present, were also killed by the antimicrobial peptides (Lewis and Shan, 2016; Vulin et al., 2018). The inability of rifampicin and ciprofloxacin to eradicate persisters is in line with other studies that showed incomplete eradication (Dunne et al., 1993; Reiter et al., 2012; Conlon et al., 2013; Molina-Manso et al., 2013; Jorgensen et al., 2016) or even induction of persisters (Kwan et al., 2013). Interestingly, rifampicin in combination with a fluoroquinolone eliminated all bacteria in several experimental animal models with foreign-body infections (Widmer et al., 1990; Zimmerli et al., 1994; Trampuz et al., 2007; Zimmerli and Sendi, 2019). The strong innate immune response in these animals may have contributed to this favorable outcome. The favorable outcome may also be related to the maturation state of the biofilms as the rifampicin combination was not effective in 2-week MRSA biofilms in a rat model (Lucet et al., 1990). In a guinea pig tissue-cage infection model rifampicin eradicated implant-adhering *S. aureus* after a 12 h treatment delay but not after a 24–48 h treatment delay (Tshefu et al., 1983). Together, results from studies on elimination of bacteria in immature biofilms in animals with a strong innate immune response may not be representative for mature biofilms in human biofilm-associated infections.

### Effectivity of Antimicrobial Peptides

The main conclusion from this study pertains to the efficacy of four promising antimicrobial peptides to eradicate persisters within antibiotics-exposed, mature biofilms. Both SAAP-148 and pexiganan rapidly eliminated biofilm-derived bacteria in a dose-dependent fashion with SAAP-148 being the most effective peptide. The required concentration of SAAP-148 to eliminate persisters within biofilms was considerably higher than for direct killing of the persisters, indicating that peptide’s antibacterial and antipersister activities are hampered by the extracellular matrix of the biofilm. Interestingly, higher SAAP-148 concentrations were needed for biofilms on TAN disks and hip liners, indicating that the surface of the implant may play a role in the development of the biofilm that protects bacteria within it. Despite displaying good antibiofilm activity in earlier studies, LL-37 reduced the bacterial counts in the antibiotics-exposed, mature biofilms only moderately (Haisma et al., 2014). ADEP4 eliminated the bacteria in the biofilms in a dose-dependent fashion at 24 h, but not at 2 h of exposure. This was expected as it takes more time before bacteria die from massive protein breakdown due to activation of the ATP-independent caseinolytic protease Clp, the proteolytic core of a major bacterial protein degradation machinery, by ADEP (Brotz-Oesterhelt et al., 2005; Conlon et al., 2016; Shan et al., 2017). Together, SAAP-148, ADEP4, LL-37, and pexiganan all exerted activity against MRSA persisters obtained from mature antibiotics-exposed, mature biofilms as well as persisters in such biofilms. The most effective peptide, SAAP-148, eliminated all persisters within mature biofilms on polystyrene, TAN disks, and on most prosthetic hip liners. Unexpected survival of bacteria was seen in the experiments with the TAN disks and in both liner experiments (Figures 5, 6). Given the high bacterial load we presumed that these bacteria had not been exposed to the antimicrobial peptide in these experiments. Therefore, we regarded those as outliers related to outgrowth of untreated persisters. Of note, SAAP-148 killed the persisters as well as log phase bacteria within 2 h, indicating that peptide’s toxic effect on bacteria is independent of the metabolic activity of bacteria. SAAP-148 kills bacteria by binding to the phospholipid bilayer of the bacterial membrane and the subsequent conformational change of the peptide that causes direct leakage of bacteria resulting in cell death.

Antibiofilm effects of SAAP-148 were evaluated on only one clinical MRSA isolate here. However, the model can be extended to the investigation of further isolates including those of clinical relevance in device-associated infections such as Coagulase-negative staphylococci and enterococci. Of note, the effectiveness of SAAP-148 against Gram-positive and Gram-negative bacteria in mature biofilms has been confirmed in another study (Nibbering et al., personal communication). SAAP-148 formulated in an ointment was also highly effective against an established biofilm-associated infection on wounded *ex vivo* human skin models (de Breij et al., 2018). Together, SAAP-148 is the most promising peptide for further development as novel agent to combat biofilm-associated infections. In order to prevent PJI, a SAAP-148 formulation may be developed as a coating for prosthetic joints and/or for application to the tissues surrounding the implant. SAAP-148 could also be used as adjunctive treatment during surgical debridement to rapidly kill any surviving bacteria after debridement.

### Other Strategies to Combat Persisters

In addition to the application of antimicrobial peptides, mechanic or enzymatic disruption of the matrix of the biofilms may also prevent the subsequent awakening of persisters rendering them susceptible to antibiotics again. Other innovative strategies, like bacteriophages and heat induction, should be further explored as viable approaches to combat clinical device-associated infections (Pijls et al., 2018; Morris et al., 2019). The biofilm model described in this study is well suited to investigate the possibilities and limitations of these strategies in more detail. Finally, combinations of various strategies may have the largest clinical effect on biofilm-associated infections.

## Conclusion

Novel *in vitro* models simulating PJI have been developed to evaluate the effects of antimicrobial peptides on persisters residing in antibiotics-exposed, mature biofilms. Combined rifampicin/ciprofloxacin treatment did not eliminate all biofilm-embedded bacteria, indicating the presence of persisters within mature biofilms. Microcalorimetry confirmed the dormant state of these bacteria. SAAP-148 eliminated persisters within mature biofilms on abiotic surfaces. SAAP-148 was more effective than LL-37, pexiganan, and ADEP4. Based on these data, SAAP-148 is a promising candidate for further development as agent to treat patients suffering from biofilm-associated infections like PJI.

## Data Availability Statement

The original contributions presented in the study are included in the article/supplementary material, further inquiries can be directed to the corresponding author/s.

## Author Contributions

HS designed the experiments and wrote draft version of the manuscript, contributed to the experiments, and integrated all comments in manuscript. JW, JV, and AV performed the most experiments and reviewed draft versions of the manuscript. LV and RW reviewed draft versions of the manuscript. MB and PHN designed experiments, reviewed draft version of the manuscript, and supervised the project. All authors contributed to the article and approved the submitted version.

## Conflict of Interest

PHN is co-inventor on patent WO-2015088344 relating to the SAAP-148 peptide studied in this paper. The remaining authors declare that the research was conducted in the absence of any commercial or financial relationships that couldbe construed as a potential conflict of interest.
